# Frequency of Gallbladder Carcinoma in Patients Operated for Symptomatic Cholelithiasis

**DOI:** 10.7759/cureus.74891

**Published:** 2024-12-01

**Authors:** Muhammad Alam, Asma Anwar, Hikmat Ullah Qureshi, Muhammad Bilawal Khan, Farah Deeba, Ali Gohar Khan

**Affiliations:** 1 Department of General Surgery, Hayatabad Medical Complex Peshawar, Peshawar, PAK; 2 Department of General Surgery, King Fahad Specialist Hospital, Tabuk, SAU; 3 Department of Public Health, Peshawar Medical Centre, Bannu, PAK; 4 Department of General Surgery, Fauji Foundation Hospital, Peshawar, PAK

**Keywords:** cholecystectomy, chronic inflammation, gallbladder carcinoma, histopathology, incidental carcinoma, symptomatic cholelithiasis

## Abstract

Background

Gallbladder carcinoma (GBC) is a rare but highly aggressive malignancy, often discovered incidentally during cholecystectomy for symptomatic cholelithiasis. Despite significant geographic variation, the association between gallstones and GBC is well-documented, with chronic inflammation from gallstones potentially contributing to carcinogenesis.

Objective

This study aims to determine the prevalence of incidental GBC in patients undergoing cholecystectomy for symptomatic cholelithiasis at a tertiary care hospital in Peshawar, Pakistan.

Materials and methods

This retrospective cohort study was conducted at the General Surgery Department, Hayatabad Medical Complex, Peshawar, from February 2, 2021, to July 1, 2024. A total of 230 patients, aged 18-55 years and diagnosed with symptomatic cholelithiasis, were included. Cholecystectomy was performed, and all excised gallbladders were histopathologically examined for carcinoma. Statistical analysis was conducted using IBM SPSS (version 26), with a significance threshold of p ≤ 0.05.

Results

Out of 230 patients, 7 (3.04%) were found to have incidental GBC upon histopathological examination. Among these, five had adenocarcinoma, and two had papillary carcinoma. The carcinoma was confined to the mucosa (T1a) in four cases, while three cases had deeper invasion (T1b). Patients with incidental carcinoma had a significantly longer symptom duration (mean: 14.2 ± 3.1 months) and were older on average (mean age: 49.2 ± 4.3 years) compared to non-carcinoma patients (p = 0.001 and p = 0.002, respectively). No malignancy was suspected intraoperatively in any case.

Conclusion

Incidental GBC was present in 3.04% of patients undergoing cholecystectomy for symptomatic cholelithiasis, with longer symptom duration and older age as significant risk factors. The findings underscore the importance of histopathological examination of cholecystectomy specimens in regions with high gallstone prevalence, as early detection of incidental carcinoma can facilitate timely oncological intervention.

## Introduction

Although gallbladder carcinoma (GBC) is among the rarest yet highly aggressive malignancies, it can be recognized incidentally in patients undergoing cholecystectomy for cholelithiasis. The global distribution of GBC shows significant geographic differences, including relatively high incidence rates in some regions (e.g., South Asia and Latin America), where gallstone disease itself is more common [1,2]. However, the association between gallstones and GBC has long been reported, with data implying that repeated injury in the form of chronic inflammation due to gallstones may play an important pathogenic role in the malignant transformation of gallbladder mucosa [3,4].

Symptomatic cholelithiasis is a common disorder that frequently requires surgical intervention, namely cholecystectomy. Incidental GBC in patients undergoing cholecystectomy has been reported with varying frequency depending on the region of the patients' origin [5]. The risk of incidental GBC in patients undergoing cholecystectomy for symptomatic cholelithiasis in high-risk areas has been reported to be between 0.2 and 2% [6,7]. Although the exact pathophysiological link between gallstones and GBC remains unclear, chronic irritation and mucosal injury due to gallstones are believed to be important risk factors for carcinoma [8].

Early detection is crucial when it comes to GBC, as these cases rarely present any symptoms until they reach an advanced stage. However, a large number of cases of GBC are diagnosed incidentally due to the nonspecific nature of the early symptoms of the disease [9]. In most cases, this is associated with a poor prognosis with high recurrence and low survival even after aggressive treatment measures. Hence, it is crucial to ascertain the incidence of GBC among patients who undergo surgery for symptomatic cholelithiasis in order to improve the efficacy of surgical management and its outcomes.

The goal of this research is to determine how common incidental GBC is in patients operated on for symptomatic cholelithiasis, thereby adding to the growing body of literature that will help in making better surgical decisions and counseling patients in regions with high gallstone disease.

## Materials and methods

This retrospective cohort study was conducted in the General Surgery Department at Hayatabad Medical Complex, Peshawar. Data was collected retrospectively by reviewing the records of 230 patients diagnosed with symptomatic cholelithiasis who underwent elective or emergency cholecystectomy between February 2, 2021, and July 1, 2024. The study included participants aged 18-55 years, diagnosed with cholelithiasis and experiencing significant pain, as confirmed through clinical examination and imaging studies like ultrasound. Prior to surgery, all patients were evaluated and deemed fit for surgical management based on their clinical history and preoperative work-up.

Several patient groups were excluded from the study: those with a preoperative or intraoperative diagnosis of GBC, those with a history of malignancy unrelated to gallbladder disease, those diagnosed with gallbladder diseases not associated with cholelithiasis such as acalculous cholecystitis or gallbladder polyps, those with comorbidities such as advanced heart failure (NYHA Class III-IV), severe chronic obstructive pulmonary disease (requiring home oxygen therapy), or end-stage renal disease (requiring dialysis) that contraindicated surgery, and those with incomplete or missing clinical and histopathological data.

All demographic and clinical data, including age, gender, presenting complaint, and duration of symptoms, were recorded. A standard preoperative routine workup was performed on all patients, including liver function tests, complete blood count, and USG of the hepatobiliary system. Additional diagnostic tools like magnetic resonance cholangiopancreatography (MRCP) and endoscopic retrograde cholangiopancreatography (ERCP) were used for further evaluation in patients with suspected biliary obstruction or choledocholithiasis. Cholecystectomies were performed using either laparoscopic or open surgical techniques, depending on clinical indications.

**Figure 1:**
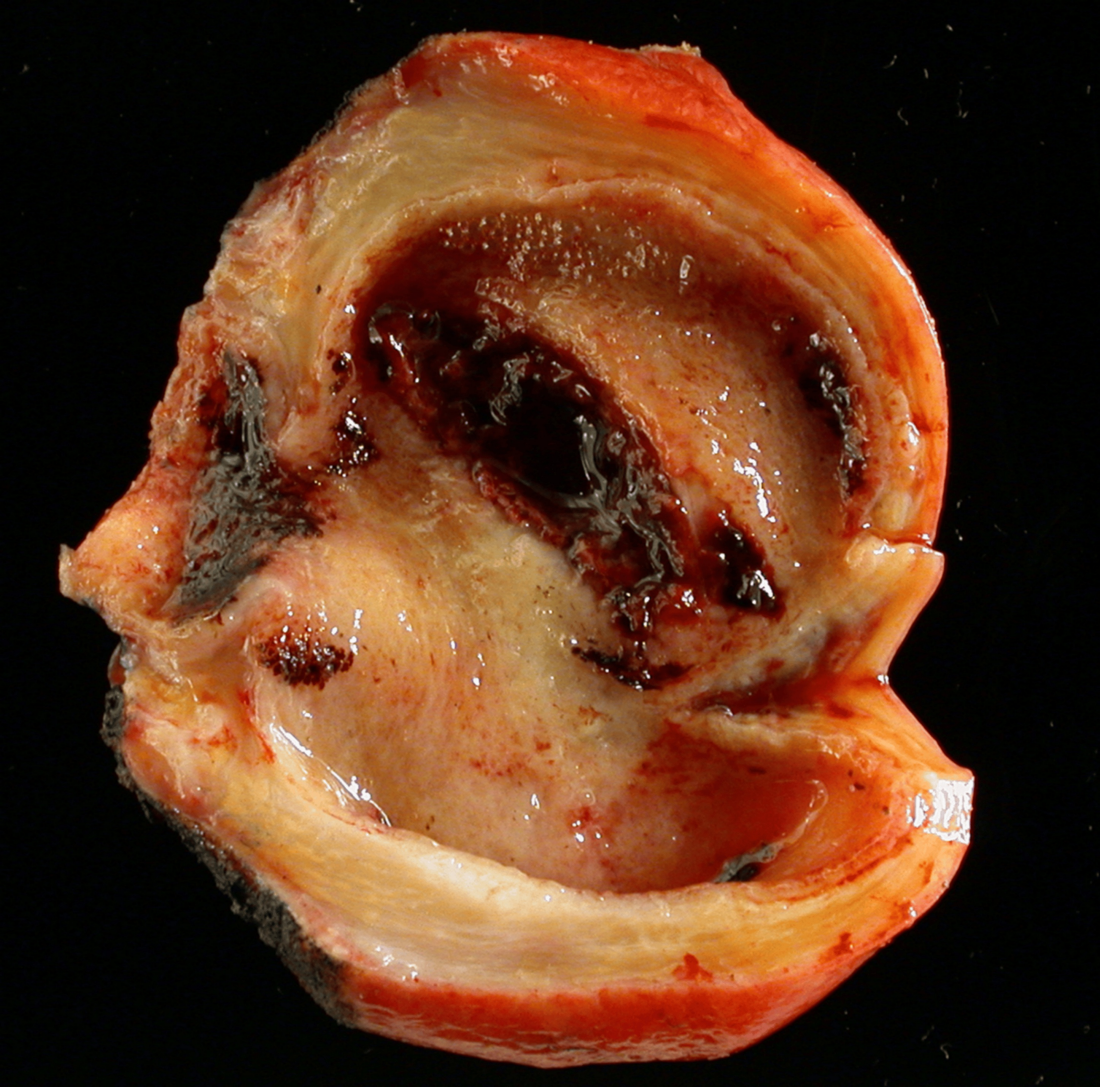
Specimen of gallbladder carcinoma.

Specimens of the gallbladder removed during surgery were sent for histopathological examination to determine the presence of carcinoma. These specimens were preserved in 10% formalin and processed using standard histological methods. Tissue sections were prepared on slides, stained with hematoxylin and eosin, and examined by a consultant pathologist to evaluate the specimen. Cases of GBC were analyzed according to histopathological type and extent of invasion as shown in Figure 1.

Data analysis was performed using IBM SPSS (version 26). Summary statistics were applied to clinical and demographic data. Age, sex, and symptom duration were examined using chi-square tests for categorical data and t-tests for quantitative data, where appropriate, in carcinoma and non-carcinoma patients. A p-value of ≤0.05 was considered statistically significant.

The study protocol was approved by the Institutional Review Board (IRB) of Hayatabad Medical Complex, Peshawar, under reference # 1679. Written consent was obtained from all enrolled patients before the operative procedure. All patient information was treated confidentially and used solely for research purposes.

## Results

A total of 230 patients who underwent cholecystectomy for symptomatic cholelithiasis at the General Surgery Department, Hayatabad Medical Complex, Peshawar, from February 2021 to July 2024, were included in the study. The mean age of the study population was 40.7 ± 9.4 years, with an age range of 18 to 55 years. Of these, 156 (67.8%) were female and 74 (32.2%) were male, resulting in a female-to-male ratio of approximately 2.1:1, consistent with the higher prevalence of gallstone disease in women.

Most patients (n=189; 82.2%) presented with right upper quadrant abdominal pain as the primary symptom. Other common symptoms included nausea (n=145; 63%), vomiting (n=98; 42.6%), and dyspepsia (n=72; 31.3%). The duration of symptoms ranged from 1 month to 2 years, with a median duration of 8 months. Of the total cases, 72 (31.3%) were acute (emergency) cholecystectomies, performed due to complications such as acute cholecystitis, empyema, or gallbladder perforation. The remaining 158 (68.7%) were chronic (elective) cholecystectomies, carried out for recurrent or persistent symptoms of gallstone disease without acute complications (Table 1).

**Table 1:** Demographics and other clinical characteristics.

Variable	n	%
Gender		
Male	74	32.2
Female	156	67.8
Symptoms		
Right upper quadrant pain	189	82.2
Nausea	145	63
Vomiting	98	42.6
Dyspepsia	72	31.3
Elective/emergency cases		
Emergency cholecystectomy	72	31.3
Elective cholecystectomy	158	68.7
Age		
Age (mean±SD)	40.7 ± 9.4	
Age (range)	18-55 yrs	
Duration of symptoms (months)		
Median duration	8 months	
Duration range	1-24 months	

Among the 230 patients, 186 (80.9%) underwent laparoscopic cholecystectomy, while the remaining 44 (19.1%) required conversion to open cholecystectomy due to intraoperative difficulties, such as dense adhesions or unclear anatomy. Preoperative radiological evaluations, primarily abdominal ultrasound, revealed thickened gallbladder walls (≥4 mm) in 152 (66.1%) patients, gallstones in all cases, and sludge in 27 (11.7%) patients. The approximate size of gallstones ranged from 0.5 cm to 2.5 cm, with a mean size of 1.3 ± 0.5 cm. Of the total cohort, 16 (7%) patients had radiological evidence of choledocholithiasis. Among these, 10 (4.3%) cases were confirmed through magnetic resonance cholangiopancreatography (MRCP), while 6 (2.6%) cases were confirmed through endoscopic retrograde cholangiopancreatography (ERCP). A total of 12 patients underwent successful ERCP with biliary stenting prior to surgery to alleviate obstruction and facilitate safe cholecystectomy. Intraoperative findings included thickened gallbladder walls, gallstones, and signs of chronic inflammation in most cases. No patients were suspected of malignancy intraoperatively based on macroscopic evaluation (Table 2).

**Table 2:** Surgical, radiological findings and interventions. ERCP: Endoscopic Retrograde Cholangiopancreatography; MRCP: Magnetic Resonance Cholangiopancreatography.

Details	Frequency (n)	Percentage (%)
Surgical approach		
Laparoscopic	186	80.9
Open	44	19.1
Radiological findings		
Thickened gallbladder walls (≥4 mm)	152	66.1
Gallstones present	230	100
Sludge	27	11.7
Stone size (mean ± SD):	1.3 ± 0.5 cm	range: 0.5-2.5 cm
Intervention		
Patients with choledocholithiasis	16	7
Confirmed via MRCP	10	4.3
Confirmed via ERCP	6	2.6
Patients who underwent ERCP	12	5.2

Postoperative histopathological examination of gallbladder specimens revealed incidental gallbladder carcinoma in 7 patients (3.04%). Of these, 5 cases were identified as adenocarcinoma, and 2 cases were diagnosed as papillary carcinoma. The carcinoma was limited to the gallbladder mucosa in 4 patients (T1a), while 3 patients had deeper invasion into the muscularis layer (T1b). None of the patients with incidental carcinoma had preoperative imaging suggestive of malignancy. The majority of incidental carcinoma cases were found in females (n=5; 71.4%), with an average age of 49.2 ± 4.3 years, older than the overall study population (Table 3).

**Table 3:** Histopathological findings.

Histopathological Findings	n	%
Incidental gallbladder carcinoma	7	3.04
Adenocarcinoma	5	71.4
Papillary carcinoma	2	28.6
Stage of carcinoma		
T1a (Mucosal invasion)	4	57.1
T1b (Muscularis invasion)	3	42.9

Patients with incidental gallbladder carcinoma had a significantly longer duration of symptoms (mean: 14.2 ± 3.1 months) compared to those without carcinoma (mean: 7.8 ± 2.7 months) (p=0.001). There was no statistically significant difference in gender distribution (p=0.712) or type of surgical approach (p=0.086) between the groups with and without carcinoma. However, older age was a significant risk factor, with patients aged over 45 years having a higher likelihood of incidental carcinoma (p=0.002) (Table 4).

**Table 4:** Association of clinical and demographic factors with gallbladder carcinoma.

Variable	Carcinoma (n=7)	No Carcinoma (n=223)	P-value
Mean age (years)	49.2 ± 4.3	40.2 ± 8.6	0.002
Symptom duration (months)	14.2 ± 3.1	7.8 ± 2.7	0.001
Gender			
Male	2 (28.6%)	72 (32.3%)	0.712
Female	5 (71.4%)	151 (67.7%)	
Surgical approach			
Laparoscopic	6 (85.7%)	180 (80.7%)	0.086
Open	1 (14.3%)	43 (19.3%)	

All patients with incidental gallbladder carcinoma were referred to the oncology department for further management. Two patients underwent re-exploration for oncological staging and extended resections. There was no postoperative mortality related to incidental carcinoma during the study period.

## Discussion

The present study intended to assess the prevalence of GBC in patients who underwent cholecystectomy due to symptomatic cholelithiasis. The incidental finding of gallbladder cancer in 7 out of 230 patients (3.04%) underscores the importance of routinely evaluating gallbladder specimens histologically, even when there is no clinical suspicion of malignancy.

Our findings align with a growing number of reports that highlight GBC as a covert condition often associated with gallstone disease, a well-recognized risk factor. The observed 3% prevalence in our study emphasizes the silent nature of this malignancy and supports the routine practice of histopathological examination of all gallbladder specimens to ensure early detection of incidental malignancies. These results are consistent with the increasing focus in the literature on the asymptomatic presentation of GBC and its notable association with gallstone disease [10,11].

We noted a female predominance in incidental GBC cases (71.4%), which aligns with published literature [12,13]. It is well established that GBC is more common in females than in males, possibly because gallstone disease is more prevalent among women due to hormones such as estrogen that help to saturate cholesterol in bile. Research from India also showed that 75% of gallbladder carcinoma patients were female, which aligns with the global gender distribution [14].

Patients with GBC in our study were older than the general population (mean age 49.2 years compared to 40.7 years). Being aged over 45 years was a significant risk factor for the presence of incidental carcinoma (p=0.002), expanding the understanding that gallbladder cancer is more rampant in the elder age group. This finding is consistent with previous reports which noted chronic gallbladder disease in elders as an independent predictor of GBC [15,16]. As a result, clinicians should maintain a high index of suspicion for malignancy in older patients undergoing cholecystectomy for benign indications.

One of the most significant findings of this study was that patients diagnosed with carcinoma had a longer duration of symptoms (14.2 months) compared to patients without carcinoma (7.8 months) (p=0.001). The reason for such prolonged symptom duration could be the delayed presentation of GBC; it has a more silent onset and resembles benign biliary disease in the early stages. Various studies have indicated that the persistent existence of gallstones may lead to chronic inflammation, which progressively leads to cancer and elucidates the link between longer symptom duration and malignancy [17,18]. Our data support this theory, and we suggest considering possible malignancy in patients who present with biliary symptoms lasting for a prolonged period.

Histopathological analysis indicates that 57.1% of incidental carcinomas were found to be T1a (resting on the surface without invading the underlying layers), while 42.9% displayed T1b characteristics (invading the layers of muscle). These early tumors usually do not produce symptoms and are therefore diagnosed asymptomatically in our study. A routine histopathological assessment of each gallbladder specimen should always be performed carefully as it is useful in revealing early cancers that would have otherwise been missed. Other studies have also demonstrated similar results, reporting that T1a and T1b carcinomas are usually found incidentally when cholecystectomy is performed for reasons other than cancer [19,20].

One of the important aspects of cancer detection at its early stage is based on various clinical findings regarding GBC. Patients with T1a disease, in which cancer is limited to the mucosa, have an excellent prognosis and potentially will not need more than a cholecystectomy. However, in T1b carcinomas with muscularis involvement, aggressive treatment, such as extended resections, may be indicated to maximize oncological safety. In this study, two patients with T1b carcinoma underwent re-exploration and extended resection, highlighting the relevance of early diagnosis and timely surgical strategy.

The frequency of incidental carcinoma (3.04%) in our study is comparatively higher than the world statistics, which usually vary between 0.2% and 2% in larger cohort studies of incidental carcinoma [21]. The causal reason may be geographical, especially since GBC is more common among South Asian countries, particularly India and Pakistan. More reports also give a glimpse of the strong link between gallstone disease and GBC, in which more than 80% of carcinoma cases are reported to be associated with cholelithiasis, a statistic that is consistent with our data [22,23]. In our study, the prevalence of adenocarcinoma (71.4%) is consistent with the histological subtype most commonly reported in gallbladder cancers worldwide.

There is also a consensus regarding risk factors such as age, sex, and duration of symptoms in our findings. Several studies report that older age, female gender, and chronic biliary symptoms are important risk factors for gallbladder cancer in patients with gallstones [24]. These data emphasize that patients with these risk factors must be monitored carefully.

It is important to point out several limitations inherent in this study. First, this study was performed at a single center, thus affecting the generalizability of the findings. Second, the retrospective design of the study was quite challenging and presented some potential risks, including reporting bias and missing data. Third, there were very few carcinoma cases (n=7), reducing the statistical power for identifying significant associations. Further studies with a larger sample size and longer prospective follow-up conducted in multi-centers are suggested to overcome these limitations in the future.

## Conclusions

The incidence of incidental GBC was 3.4% among patients who underwent cholecystectomy for symptomatic cholelithiasis. While the routine histopathological examination of gallbladder specimens is widely recognized as standard practice globally, our findings emphasize its continued importance, particularly in regions with a high prevalence of gallstone disease. This practice not only aids in identifying occult malignancies but also facilitates early intervention, significantly impacting patient outcomes. The predominance of GBC in older females, along with a notable association with prolonged symptom duration, highlights the importance of thorough evaluation in at-risk populations. These results underscore the necessity for heightened clinical awareness and further research to improve early detection strategies for GBC.
